# Linking frass and insect phenology to optimize annual forest defoliation estimation

**DOI:** 10.1016/j.mex.2023.102075

**Published:** 2023-02-15

**Authors:** B. Thapa, P.T. Wolter, B.R. Sturtevant, J.R. Foster, P.A. Townsend

**Affiliations:** a Department of Natural Resource Ecology & Management, Iowa State University, Ames, IA 50011, USA; b Institute for Applied Ecosystem Studies, Northern Research Station, USDA Forest Service, Rhinelander, WI 54501, USA; c Rubenstein School of Environment and Natural Resources, University of Vermont, Burlington, VT 05405, USA; d Department of Forest and Wildlife Ecology, University of Wisconsin—Madison, 1630 Linden Drive, Madison, WI 53706, USA

**Keywords:** *Choristoneura pinus* F*.*, *Lymantria dispar dispar* L*.*, BioSIM, Optimization, Imputation, Frass, Foliage loss, Annual defoliation, Estimation of annual defoliation based on field-collected frass and insect phenology

## Abstract

It is often logistically impractical to measure forest defoliation events in the field due to seasonal variability in larval feeding phenology (e.g., start, peak, and end) in any given year. As such, field data collections are either incomplete or at coarse temporal resolutions, both of which result in inaccurate estimation of annual defoliation (frass or foliage loss). Using *Choristoneura pinus* F. and *Lymantria dispar dispar* L., we present a novel approach that leverages a weather-driven insect simulation model (BioSIM) and defoliation field data. Our approach includes optimization of a weighting parameter (w) for each instar and imputation of defoliation. Results show a negative skew in this weighting parameter, where the second to last instar in a season exhibits the maximum consumption and provides better estimates of annual frass and foliage biomass loss where sampling data gaps exist. Respective cross-validation RMSE (and normalized RMSE) results for *C. pinus* and *L. dispar dispar* are 77.53 kg·ha^−1^ (0.16) and 38.24 kg·ha^−1^ (0.02) for frass and 74.85 kg·ha^−1^ (0.10) and 47.77 kg·ha^−1^ (0.02) for foliage biomass loss imputation. Our method provides better estimates for ecosystem studies that leverage remote sensing data to scale defoliation rates from the field to broader landscapes and regions.

• Utilize fine temporal resolution insect life cycle data derived from weather-driven insect simulation model (BioSIM) to bridge critical gaps in coarse temporal resolution defoliation field data.
• Fitting distributions to optimize the instar weighting parameter (w) and impute frass and foliage biomass loss based on a cumulative density function (CDF).
• Enables accurate estimation of annual defoliation impacts on ecosystems across multiple insect taxa that exhibit distinct but seasonally variable feeding phenology.

## Graphical abstract

**Figure:**
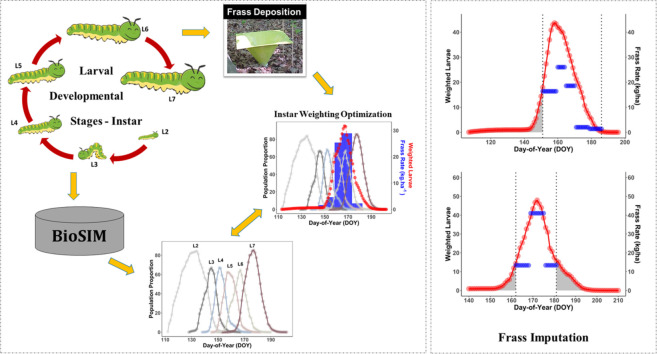

Specification table

**Table:** 

Subject area:	Agricultural and Biological Sciences
More specific subject area:	Insect disturbance
Method name:	Estimation of annual defoliation based on field-collected frass and insect phenology
Name and reference of original method:	NA
Resource availability:	https://github.com/ThapaBina/SeasonalDefoliation_Estimation

## Introduction

In the past two decades, insect disturbance and associated impacts on forest ecosystems have gained increasing attention due to the uncertain or variable response dynamics associated with climate forcing [44]. Notably, carbon fluxes and subsequent impacts on net primary production (NPP) and net ecosystem productivity (NEP) from biomass loss to insects are major concerns [14,30,55]. For instance, Kurz et al. [30] reported maximum average (mode) annual emission at 100 Mt CO_2_ yr^−1^ from insect disturbances in boreal forest. Similar observation was reported by Dymond et al. [14], where *Choristoneura fumiferana* Clem caused a reduction of 2 Mt C yr-^1^ in net primary production in boreal forests via a combination of growth reduction and mortality. Both growth reduction and tree mortality are functions of the cumulative annual loss of foliage (photosynthetic material) [7,19,29]. As such, leaf area loss is a principle component of defoliation impact assessment for economically important defoliators such as *C. fumiferana, Choristoneura pinus* F., *Malacosoma disstria* Hübner, and *Lymantria dispar dispar* L. [11,35]. In turn, annual loss of foliage affects both net primary productivity and nutrient fluxes of a forest ecosystem [8,21,27,36,51]. Therefore, it is important to accurately estimate total foliar biomass loss to better quantify impacts of various defoliators on forest ecosystems.

Foliage biomass consumption by many of the economically important defoliators of North America (including all listed above) occurs in spring season as early stage larvae either hatch or emerge from winter diapause to feed on newly emerging nutrient-rich and poorly-defended foliage [9]. Timing of emergence in these species is essential for their survival; if emergence is too early larvae starve for lack of food, and if too late larvae will encounter increasingly defended food resources [20]. As a larva feeds and gains biomass, it periodically molts its head capsule into its next development stage of larvae or “instar,” where the number of instars varies by species [13]. With each molt, the mouth size of the larva increases, which results in an increase in food intake and, thus, body size [4,22,24,41]. The rate of food intake increases exponentially across the final stages of larval development [28,39]. For example, Miller [39] found that the fifth– and sixth (or final) instars of *C. fumiferana* consumed 9% and 87% of total host foliage, respectively. Past research has linked species-specific instar phenology to specific weather events (e.g., [48]), which may be used to project the timing of successive larval instars of select forest insect defoliators in time and space [46].

In this study, we analyzed frass (excrement) deposition data collected during late spring and early summer seasons of outbreak years (2000, 2001, 2006 and 2007 for *L. dispar dispar* and 2006–2007 for *C. pinus*) to estimate annual biomass loss due to defoliation. Contrary to traditional field measures of annual defoliation [e.g., shoot method [16]], frass is directly associated with the caterpillar feeding biology and, when scaled by instar feeding efficiency, represents the amount of foliage consumed [50]. In addition, analysis of daily frass deposition collected throughout a feeding season can confirm instar phenology [3]. Thus, the amount of frass deposition at a given time is a function of total amount of food consumed by individuals of different stages of instar of respective defoliators and density of instars [3,33,50]. However, variability in the start and end of insect feeding in a given year, and from site to site, complicates field sampling efforts, which affects our ability to accurately quantify total frass deposition, especially when it is unknown where and even if a defoliation event is forthcoming. For example, frass collection starting either too late or ending too early leads to under estimation of total frass deposition for a season. In addition, limited resource availability affects the temporal resolution of field data collection during the feeding season, such as sampling frequency (total number of days or weeks) and frass collection periods (hourly, daily, or weekly).

Here, we present a novel approach to estimate annual frass deposition and biomass loss. This is achieved by utilizing frass and greenfall deposition data (kg⋅ha^−1^) measured in the field at coarse temporal resolution and simulated fine temporal resolution (daily) population phenology data (BioSIM V.11, [46]) for two major economically important spring defoliators: *L. dispar dispar* (non-native) and *C. pinus* (native). The exotic moth *L. dispar dispar* is a broad-leaf defoliator that prefers oak (*Quercus* spp.), but also feeds on other broad-leaf tree species [32]. The endemic moth *C. pinus* is a selective, conifer defoliator that feeds only on jack pine (*P. banksiana*) pollen cones and foliage [37].

This approach includes a two-step process, where the first step is estimation and optimization of an instar weighting (w) parameter for respective defoliators, representing the relative amount of foliage removed during a given instar life stage. The second step involves imputation of frass biomass for missing days (day-of-year [DOY]) within the respective defoliator's phenological feeding window within the field-sampling period.

Combining different data types is common practice among species distribution modeling efforts to improve presence/absence model accuracies [17,40]. However, to our knowledge, this is the first study to demonstrate the integration of different data types (i.e., temporal resolution: fine vs coarse, source data: field vs simulated, and measurement unit: kg vs proportion) based on feeding phenology of defoliators. Here, it is important to note that ‘biomass’ is used for frass and foliage loss to quantify the total amount of mass and is expressed in kilograms (kg). However, instar ‘weighting (w)’ is applied in the statistical sense of a coefficient between zero and one reflecting the relative contribution of a given instar to frass deposition or foliage loss (collectively, metrics of foliage consumption) during the entire feeding period. Annual defoliation estimates i.e., annual frass biomass is defined as the cumulative annual frass deposition during the entire feeding period (season) of each respective defoliator in any outbreak year. We further extended the utility of our approach by estimating leaf biomass (kg) consumed by caterpillars of *L. dispar dispar* and *C. pinus* from frass deposition rates via digestibility parameters derived from literature-reported laboratory food use efficiency trials, and then calculated the foliage biomass loss for this paper. This method also provides an opportunity to utilize the vast amount of existing data collected in the past for better understanding of ecological process. Finally, annual estimates of defoliation from this approach will be useful to assess the nutrient fluxes via frass biomass and carbon dynamics via foliage loss when translated to growth reduction via empirical functions, which is scalable to landscape levels via remote sensing data [49,51].

## Materials and methods

### Study area and data collection

**Fig. 1:**
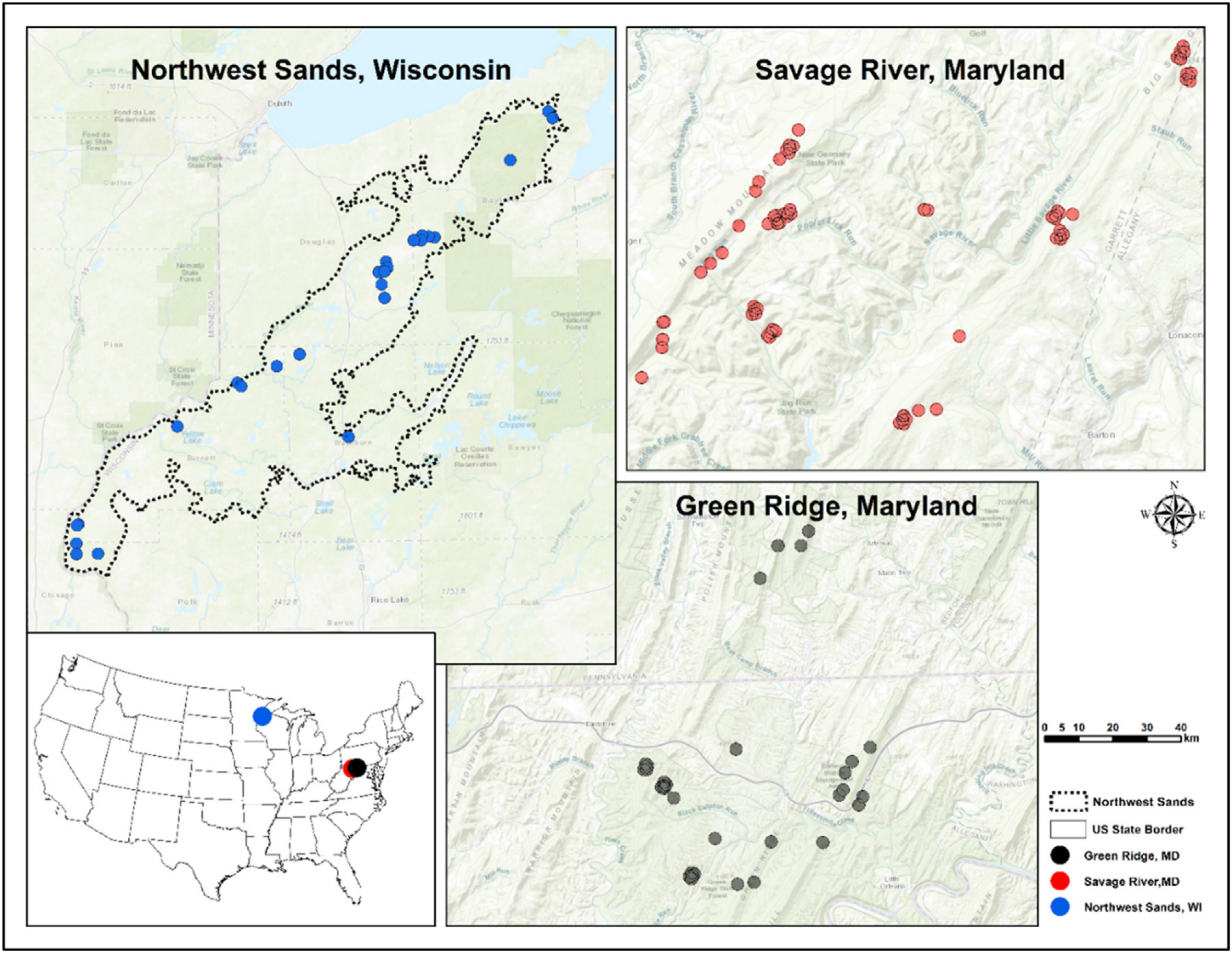
Location of study sites of *C. pinus* in Northwest Sands, Wisconsin (blue) and *L. dispar dispar* in Green Ridge (GR) (black) and Savage River (SR) (red ) in Maryland in USA.

We collected *L. dispar dispar* frass deposition from western Maryland for outbreak years 2000–2001 and 2006–2007 and *C. pinus* frass deposition from northern Wisconsin (Northwest Sands region) for 2005–2006 (Fig.1, Table 1). Mixed deciduous forest with a predominance of oak species (*Quercus* spp.) characterize the Maryland sites (Green Ridge [GR] and Savage River [SR]), where less abundant tree species include maples (*Acer* spp.*)*, cherry (*Prunus* spp.*)*, ashes (*Fraxinus* spp.), and eastern white pine (*Pinus strobus*) [18,52]. Forests within the Northwest Sands region of Wisconsin are comprised of both naturally occurring jack pine stands and plantations of jack pine (*Pinus banksiana*), red pine (*Pinus resinosa*), and eastern white pine (*P. strobus*), with a predominance of jack pine. Broadleaf tree species also occur in mixture with native conifer stands such as northern pin oak (*Quercus ellipsoidalis* E.J. Hill), aspen (*Populus* spp.) and paper birch (*Betula papyrifera* Marsh), which represent ca. eighteen percent of the forested area [54].

**Table 1:** Total number of sample size used to collect the frass data of each defoliator in each year with field season starting and ending day.

Defoliators	Year	Collection Day		Total plots (n)
		Start	End	
*L. dispar dispar*	2000	31-May	28-Jul	33
	2001	17-May	12-Jul	12
	2006	30-May	12-Jul	20
	2007	22-May	5-Jul	20
*C. pinus*	2005	14-Jun	7-Jul	15
	2006	31-May	8-Jul	21

The primary local feeding periods in the Maryland and Wisconsin study locations are similar, where both *L. dispar dispar* and *C. pinus* feed from mid-May to early-July [1,45]. However, their overwintering strategies differ, as *L. dispar dispar* overwinters in the egg stage [25], while *C. pinus* hatch in late summer and almost immediately molt into their second instar (i.e., L2), after which they form a hibernaculum to overwinter in diapause (i.e., arrested development) [47]. In western Maryland, *L. dispar dispar* larvae typically hatch from eggs anywhere between mid-April and early May depending on weather [20,45]. In Northern Wisconsin, *C. pinus* break diapause in early May [1]. For both species, total foliar biomass loss consists of direct consumption and unconsumed foliage that becomes severed during feeding. The latter component of foliar loss, referred to as “greenfall,” can comprise a significant proportion of total defoliation by either species.

We established a total of 26 and 65 variable-radius plots for *C. pinus* in Wisconsin and *L. dispar dispar* in Maryland, respectively (Table 1). Each plot consisted of a central subplot and four satellite subplots arranged in the cardinal directions from plot center, with a distance of 25 m (*C. pinus*) and 30 m (*L. dispar dispar*) between the center subplot and each satellite subplot (Appendix S: Figure S1). Traps used were either Tullgren funnels or laundry baskets fitted with cotton sheets to create a funnel porous to water and varied in shape (rectangular, circular, and cone), and size (range of surface area: 1231.63 – 2370 cm^2^). Four to six frass traps in each *C. pinus* plot were placed in the outer one-third of the canopy footprint for host trees randomly selected from subplot canopy trees sampled using a metric factor 2 Bitterlich prism [5], one in each satellite subplot and two in the center subplot. Frass was collected continuously for approximately 7-day intervals. In Maryland, sampling weekly *L. dispar dispar* frass deposition involved setting up four to five traps in the center plot, with frass traps set for approximately 24 hours in most cases. Location for these traps were one-half to two-third of the distance between the stem and crown border of randomly selected host trees (as above). Deposited material was sorted in the lab to separate green foliage (i.e., greenfall) from frass, sieved to remove other detritus, and then oven dried (both frass and greenfall) at 55 °C for a minimum of 72 hr. We scaled the dry mass of frass and greenfall to kg⋅ha^−1^ based on size of frass traps [33], and then converted to daily rate (kg⋅ha^−1^⋅day^−1^) by assuming an average deposition rate over the period of sampling (nearest minute) for each respective sample, and then averaging frass and greenfall deposition rates across frass traps to derive plot-level deposition rates.

After estimating dry frass biomass (kg·ha^−1^) from field data, we used this value to estimate actual amount of foliage consumed by larvae. As the amount of frass biomass production depends on the approximate digestibility (AD) of larvae of respective defoliators, we first estimated the actual amount of foliage consumed (i.e., ingested biomass, IB) by larvae (Eq. (1)). The AD is the proportion of ingested host biomass dedicated to growth of the larva (or energy expenditure) that does not become frass and varies with caterpillar species. In this case, the AD of host foliage for *C. pinus* was estimated as 0.30 based on study of the closely-related spruce budworm (*C. fumiferana*) [6] and 0.35 for *L. dispar dispar* [2].

(1) $$\text{Ingested}\;\text{biomass}\;\left(\text{IB},\frac{\text{kg}}{\text{ha}}\right)=\;\frac{\text{frass}\;\left(\frac{\text{kg}}{\text{ha}}\right)}{1-\text{AD}}$$

We then added greenfall biomass (kg·ha^−1^) collected in frass traps to estimated ingested biomass (IB) from Eq. (1) to calculate total foliage biomass loss (kg·ha^−1^) and converted to foliage biomass loss per day (kg·ha^−1^·day^−1^), similar to frass rate.

We obtained respective caterpillar phenologies from BioSIM (V.11), which incorporates weather patterns to simulate the emergence of moth, egg hatch, larval instars, and pupae (i.e., life stages). BioSIM interpolates weather station data according to plot latitude and longitude to estimate population percent within each respective life stage for each day-of-year (DOY) corresponding to respective outbreak years of field data collection. As such, BioSIM estimates the relative proportion of a population at a given life stage at a particular time and place, and not the total size or density of the population. For our purposes, we only considered larval instars relevant to the spring-summer feeding season. Feeding population percent at any given day of year (DOY) and location is the proportion of any individual instars of the respective defoliator's caterpillar, whose values range from 0 to 100 (Appendix S: Figure S2). The maximum number of larval instars, hereafter referred to as instars, can vary by species and sex [13,15,24,41]. Here, *C. pinus* has a maximum of seven instars (male and female), while *L. dispar dispar* has a maximum of five (male) and six (female) instars (Appendix S: Figure S2).

### Instar weighting optimization

Nutritional needs and foliar consumption are proportional to instar size. The amount of food consumed by the first instar (L1) for either species is negligible, so we used the second instar (L2) through the last instar for each defoliator to optimize the instar weighting parameters: *L. dispar dispar* instars L2-L6 and *C. pinus* instars L2-L7 (Appendix S: Figure S2). Instar weighting (w) approximates the relative amount of food consumed by each instar, where the value of ‘w’ is always greater than zero and the sum of instar weighting parameters represents total seasonal consumption and is equal to one. Relative amount of food consumed by an instar can be determined by either using actual amount of food eaten or frass [3]. Using the instar weighting parameters and population proportions for each instar at any given DOY, we calculated for each date a total feeding proportion, calculated simply as the sum of w times population proportion (hereafter “weighted larvae”). Weighted larvae at any given time are correlated with field-collected frass data on the same date (Appendix S: Figure S3).

We optimized instar weighting (w) by iteratively re-fitting a distribution function to the feeding population percent and field-collected frass data (Box 1). First, we fitted a distribution function to daily frass data (frass rate, kg·ha^−1^·day^−1^) such that root-mean-square-error (RMSE) between fitted frass and observed frass was low. We then initialized the model with arbitrary instar weighting (w) to calculate the weighted larvae and fitted a distribution to it. The process of fitting distribution function to weighted larvae continued until the difference between field-collected frass and fitted weighted larvae was minimized. To calibrate the model, we selected plots (12 *C. pinus* plots from 2006 and 12 *L. dispar dispar* plots from 2007) that had best captured the phenology of caterpillar's feeding behaviors (i.e., low initial frass deposition rate in spring and maximum deposition proximal to the middle of the season). BioSIM-derived feeding population percent for each developmental stage for these plots was linked to field-collected frass data based on DOY. We used leave-one-out cross validation (LOOCV) method and reported root mean squared error (RMSE) and normalized RMSE (nRMSE = RMSE/mean, [38]). Here, we determined the instar weightings of respective caterpillars by using separate models for field-collected frass as well as foliage biomass loss.

Box 1 **Instar Weighting Optimization**

Alt-text: Unlabelled box

### Imputation based on cumulative density function

**Fig. 2:**
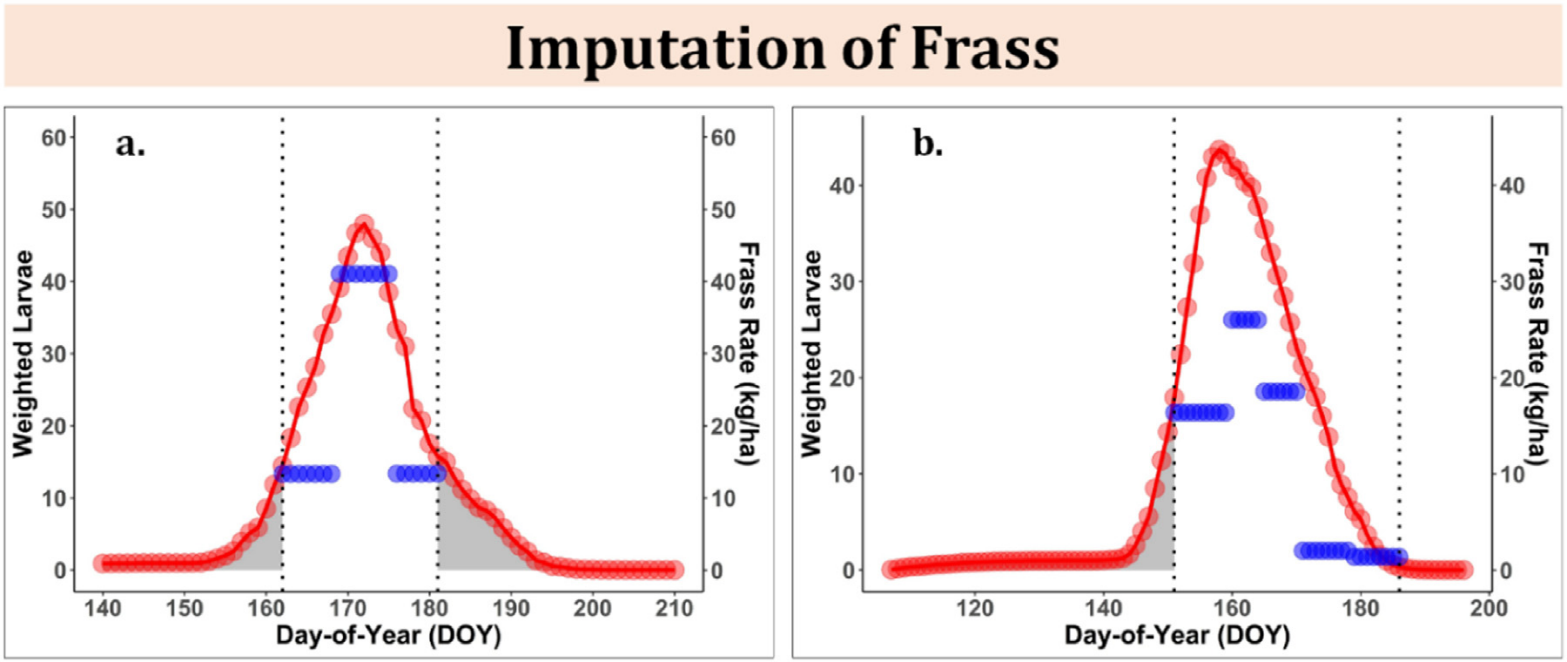
Field-collected frass data (blue) and estimated weighted larvae data (red) overlaid according to common day-of-year (DOY) for two example collections of *C. pinus*: a) beginning and end of season are un-sampled, and b) beginning of season is missed. Average frass rates were applied during each “weekly” collection period for this species (*C. pinus*). Weighted larvae is the sum of the percent of instars feeding (data obtained from BioSIM V.11) scaled by instar weightings. gray shaded areas represent missing frass data due to mismatch in field data collection start and end dates and BioSIM generated weighted larvae percentages. The vertical dotted lines represent start and end dates of field frass collection. The red line represents the cumulative density function of weighted larvae percentage used to impute the frass in missing field data collection.

We utilized the respective caterpillar phenological proportions by instar from BioSIM and instar weighting parameters obtained from the optimization process to estimate weighted larvae for each DOY. We calculated a probability distribution curve of weighted instar for each DOY and used this information to estimate either frass or foliage biomass loss for missing days in feeding phenological window during the field data collection. To illustrate, we show two examples of *C. pinus* in which we imputed frass data for two periods before DOY 162 and after DOY 181 (Fig. 2a), while only one period before DOY 151 (Fig. 2b). We validated this approach by using a K-week cross validation procedure in which we withheld one week of frass data (test data) for each plot and then estimated the cumulative frass biomass based on the remaining data as linked to weighted instar. We then calculated the predicted frass biomass for the withheld week by taking the difference between cumulative frass biomass based on all data and frass biomass using the training data. We calculated the RMSE and nRMSE of the biomass estimate using the predicted and observed frass biomass for all plots that used in the instar weighting optimization. Finally, we estimated annual frass biomass, which is the cumulative frass biomass (kg·ha^−1^) for each plot for each year by summing over daily frass rate for the entire feeding season. We repeated the same process for *L. dispar dispar* frass, as well as foliage biomass loss to *L. dispar dispar* and *C. pinus*.

## Results

**Fig. 3:**
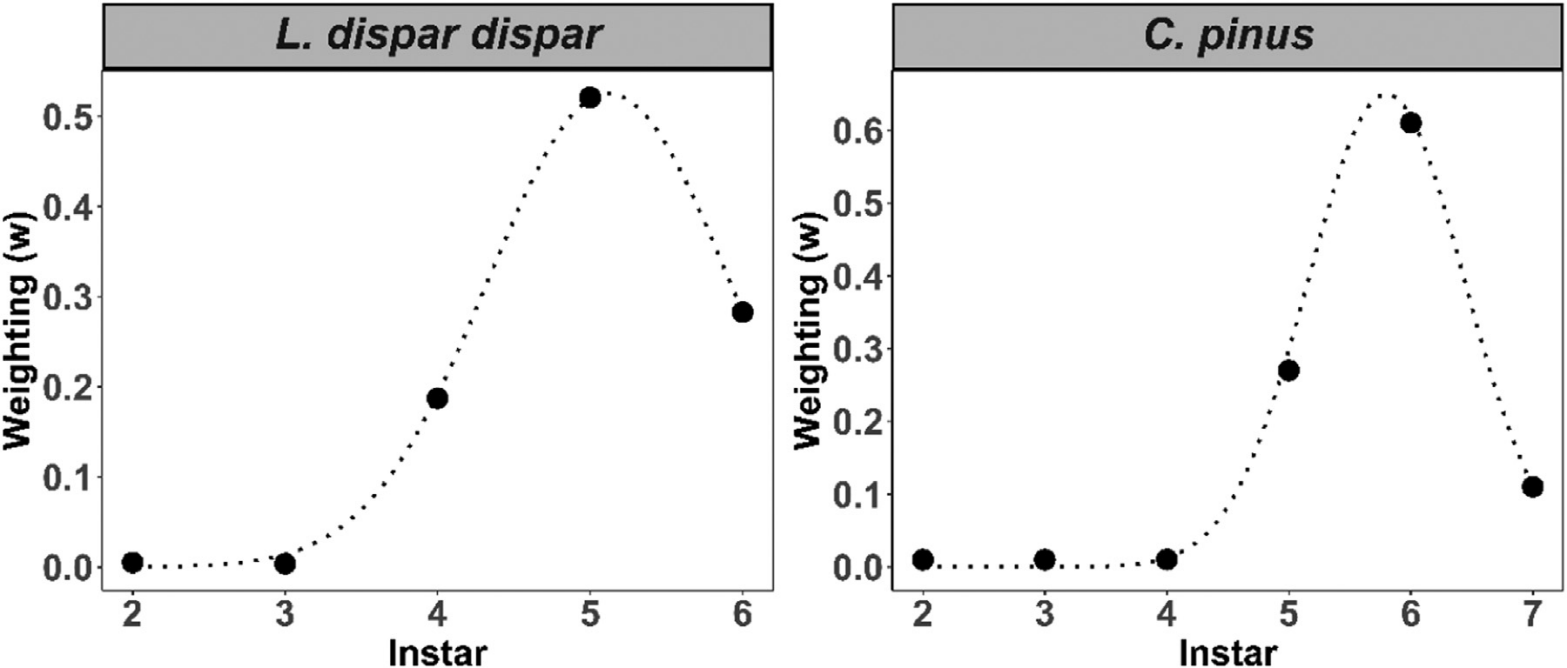
The distributions of optimal weighting parameters (w) for defoliator instars (black circle): *C. pinus* (right) and *L. dispar dispar* (left). A Non-linear distribution function in the form *y* = a * exp(-(0.5*((x-b)/c)^2)) is fitted to instar weightings of the respective defoliators (black dotted line). Higher weightings are assigned to late-stage instars because weighting is proportional to the relative amount of foliage consumed.

**Fig. 4:**
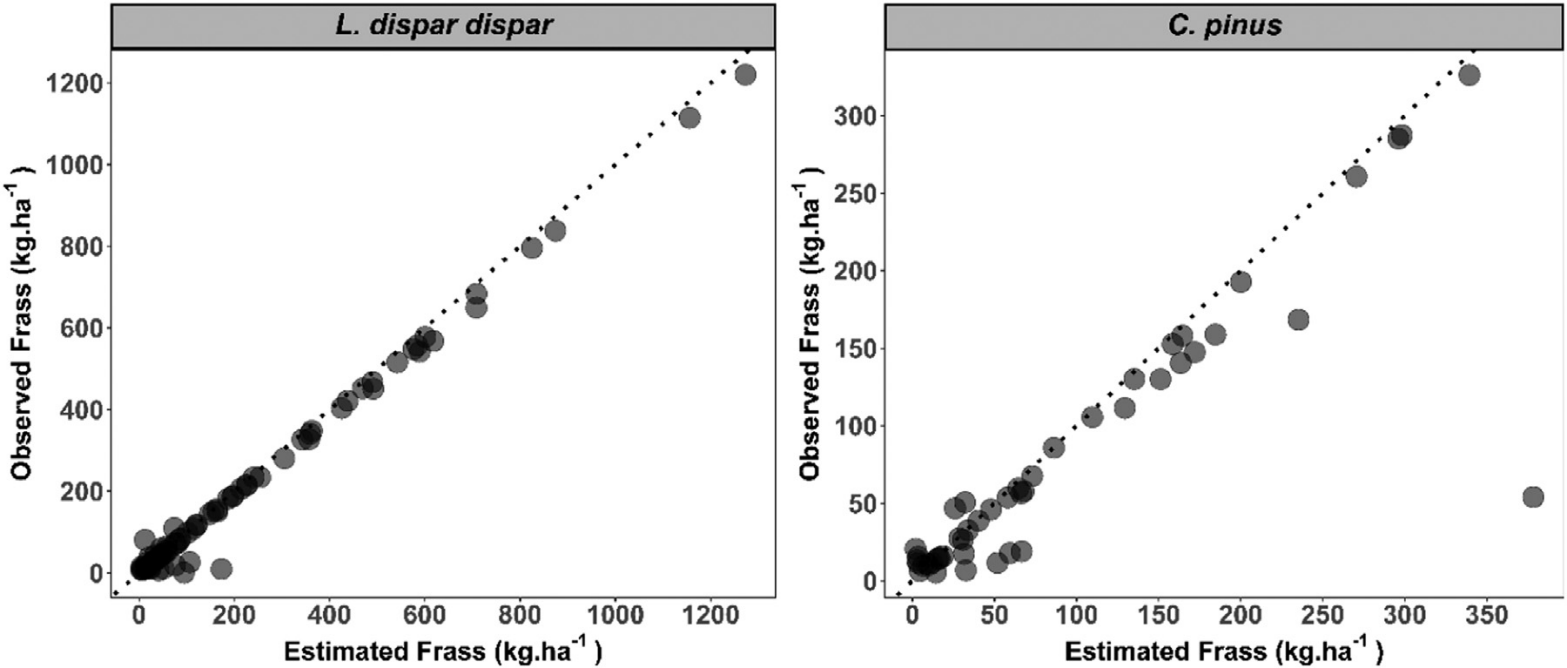
K-week cross validation results for *L. dispar dispar* (left) and *C. pinus* (right). Cross-validation was conducted to test frass data imputation model accuracy for missing days in the feeding phenological window during field data collection of each year. Imputation was conducted based on a cumulative density function of weighted instar percent in which one week field-collected frass data was held out for validation (Y-axis). Remaining data were used to estimate the frass biomass for both the week of validation and the feeding season for each year (x-axis).

**Fig. 5:**
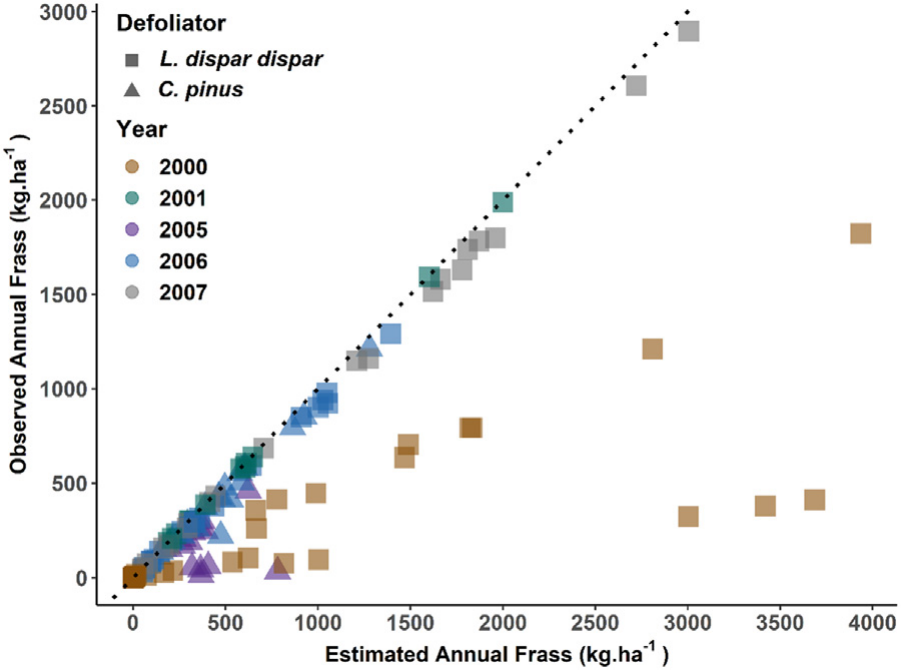
Plot of observed vs. estimated annual frass for each plot of *C. pinus* and *L. dispar dispar*. Here, annual frass data for each year is the sum of the daily frass data within the feeding season for the respective defoliators. Estimated annual frass (x-axis) represents the total of field-collected frass data and imputed frass data for missing days in the feeding phenological window based on BioSIM (V.11) population percent data. Observed annual frass data for respective years represents the sum of field-collected frass data over the season. Field-collected frass data captured the phenology of feeding population percent in most cases except for *L. dispar dispar* for the year 2000.

The distribution of instar weightings showed a negative skew, where the proportion of food consumption was low during earlier instars and increased exponentially until the second to last instar (Fig. 3). For instance, maximum feeding occurred at the sixth instar (*w* = 0.61) and fifth instar (*w* = 0.52) for *C. pinus* and *L. dispar dispar*, respectively. After their respective peak feeding, *L. dispar dispar* continued to feed on foliage (*w* = 0.28 at L6) at their final stage, while *C. pinus* showed a decrease in feeding. For instance, the *C. pinus* L7 stage had a lower relative weighting than its L5 stage (i.e., L5 [*w* = 0.29] > L7 [*w* = 0.11], Fig. 3). Cross-validation RMSE of frass biomass obtained during the optimization process for *C. pinus* were comparatively lower than *L. dispar dispar* (14 kg·ha^−1^ vs 34 kg·ha^−1^), which when normalized by mean (nRMSE) were equivalent to 0.02 for each defoliator. However, K-week cross-validation results of frass for imputation based on a probability density function of weighted larvae were opposite, where *C. pinus* had higher RMSE (and nRMSE) than *L. dispar dispar* (77.5 kg·ha^−1^[0.16] vs 38 kg·ha^−1^[0.02]) (Fig. 4). We, then, estimated annual frass biomass for both caterpillar species (Appendix S: Figure S4) and compared these data with actual field data (Fig. 5). This approach compensated the underestimation of frass biomass from the field data especially for *L. dispar dispar* in 2000 and *C. pinus* in 2005. We found that the average annual frass biomass for *C. pinus* was 377 kg·ha^−1^ for 2005 and 452 kg·ha^−1^ for 2006. Similarly, average annual frass biomass estimates for *L. dispar dispar* were 911 kg·ha^−1^, 641 kg·ha^−1^, 486 kg·ha^−1^, and 1080 kg·ha^−1^ for 2000, 2001, 2006 and 2007, respectively.

**Fig. 6:**
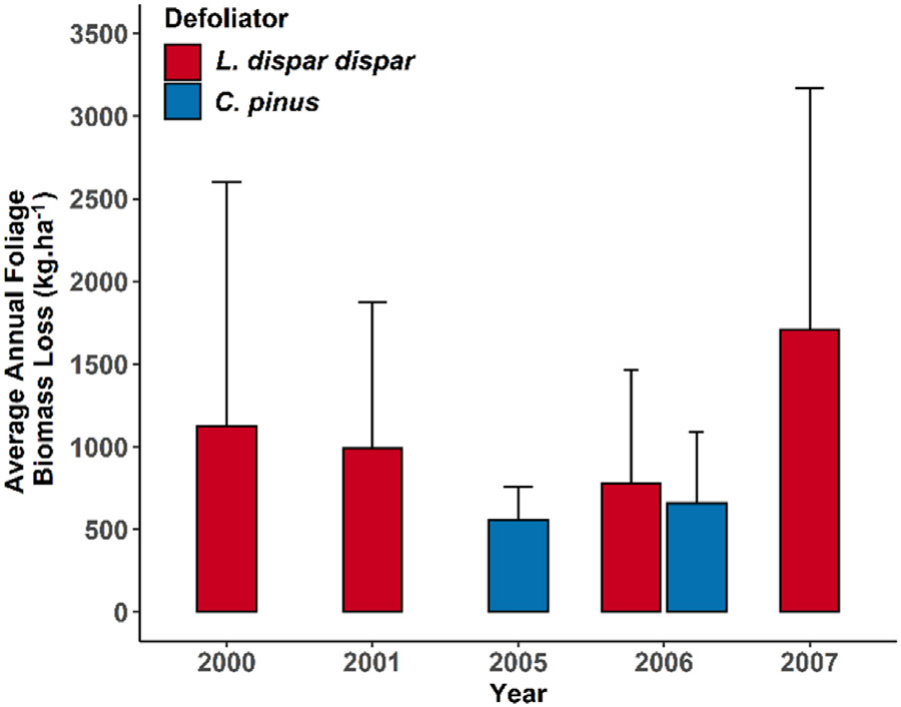
Distribution of average annual foliage biomass loss (kg·ha^−1^) by year estimated from instar weightings and then imputed for missing days in the feeding phenological window derived via BioSIM (V.11) for *C. pinus* (blue) in Wisconsin and *L. dispar dispar* (red) in Maryland. Error bars represent one standard deviation of annual foliage biomass loss.

We found similar patterns of instar weighting for each caterpillar when we used foliage biomass loss with different weightings parameters (Table 2). For instance, *C. pinus* had a maximum weighting at L6 (*w* = 0.69) and instars before (L5) and after the peak (L7) obtained equal weightings (*w* = 0.13). However, the last two *L. dispar dispar* instars obtained approximately similar weightings (*w* = 0.42 and 0.41 for L5 and L6, respectively), which accounted for almost 83% of foliage biomass loss due to defoliator. Biomass cross validation RMSE (nRMSE) values for *C. pinus* were 17.1 kg·ha^−1^ (0.02) and 74.8 kg·ha^−1^ (0.10) for the optimization and imputation, respectively. Similarly, biomass cross validation RMSE (and nRMSE) values for *L. dispar dispar* models were 57.4 kg·ha^−1^ (0.02) and 47.7 kg·ha^−1^ (0.02), respectively, for optimization and imputation. Results indicate that the average annual biomass loss in *L. dispar dispar* sites was substantially higher than in *C. pinus* sites (Fig. 6). Maximum annual foliar biomass loss to *C. pinus* occurred in 2006 (658 kg⋅ha^−1^). In the case of *L. dispar dispar*, the maximum annual foliar biomass loss across the years of the study was in 2007 (1706 kg⋅ha^−1^) followed by 2000 (1128 kg⋅ha^−1^) (Fig. 6).

**Table 2:** Optimal weighting of individual instars of respective defoliators obtained based on the feeding phenology of developmental stages of defoliators (BioSIM V. 11) and field-collected frass data (kg⋅ha^−1^) over the feeding season.

Defoliators	Instars					
	L2	L3	L4	L5	L6	L7
*C. pinus*	0.0099	0.0098	0.01	0.1399	0.6917	0.1387
*L. dispar dispar*	0.0068	0.0068	0.1524	0.4218	0.4122	NA

## Discussion

Rate and amount of foliage consumption by defoliating lepidopterans is clearly associated with the development stage of caterpillars. Our results show that instar weighting distribution estimated from modeled relative population size and frass collection is distinctly non-linear across a given defoliation season. Instar population weighting is relatively low among early instars and substantially higher for the later instars, as food consumption increases exponentially. This general relationship between food consumption rate and later instar developmental stage is evident in other studies [28,34,53]. For instance, Kulman and Hodson [28] reported most foliage consumption occurred in last two instars of *C. pinus*. However, our *C. pinus* results indicate an increase in food consumption at L5, maximum consumption at L6, followed by decreasing consumption at L7. This pattern of food consumption is also reported in previous studies of *C. fumiferana*, which has a similar life history to that of *C. pinus*. For instance, Miller [39] reported an increase in food consumption rate from 9% to 87% for L5 and L6 instars, respectively. We suspect a big drop in instar weighting at L7 is influenced by the proportion of L6 instar that survive to the next developmental stage [31,41]. The total number of instars in *C. pinus* depends on whether parasitoids have affected larvae or not. For instance, Nealis [41] showed that *C. pinus* without parasitoids exhibited seven instars while parasitoid affected *C. pinus* had six instars. In addition, parasitism rates at late instars can be high when the *C. pinus* population is at outbreak levels [42].

Unlike in *C. pinus*, we did not observe a sharp decline in frass deposition rates with the final instar of *L. dispar dispar* (L6). Importantly, half the *L. dispar dispar* population (i.e., males) complete larval development at L5, so the instar weighting for L6 in this species is applied to a proportion representing half of the insect population at that time. Leonard [34] showed that 90% of the total frass biomass produced in a season came from the last two instars, while 65% came from the last instar alone. When we use foliage biomass loss, we observe a similar pattern with respect to different instar weightings (Table 2). For instance, the L5 and L6 instars have approximately equal weighting of 0.41, while the L4 instar has 0.15. In any event, our results show that the second to last instar has a relatively high food consumption rate and the distribution of weightings within respective instars appears to corroborate finding from prior studies [28,34,39,53].

Variation in the instar weighting parameters, particularly at later instars, may be related to multiple factors. BioSIM estimates the proportion of a population at a given life stage but cannot estimate the population size of those life stages. Life stage population size can be affected by stage-dependent dispersal, parasitism, or predation rates that are further affected by outbreak phase (start, peak, and decline) and weather anomalies (e.g., late frosts). For instance, later instars are large enough to be visible to birds - making them vulnerable to predation [10,12]. Similarly, low pollen production followed by heavy defoliation in *P. banksiana* affects the survival of second instar in the spring and, consequently, the population density [31,43]. Likewise, field-collected frass may represent less than actual consumption, especially in *C. pinus* because both frass and greenfall may be temporarily retained in the canopy via their silken webs. Such variability suggests that the methods applied in this paper should be applied within the context of a specific field study to account for taxonomic and population differences across studies.

Our approach addresses uncertainties in annual estimates of defoliation due to temporal sampling mismatches with key feeding phenologies (e.g., start, peak, and end) through the feeding season. Depending on the timing of field data collection relative to phenological feeding period of larvae, average seasonal difference between actual and estimated frass was negligible for *L. dispar dispar* in 2001 (average = 7.4 kg·ha^−1^, standard deviation = 5.8 kg·ha^−1^) but large in 2000 (average = 637 kg·ha^−1^, standard deviation =915 kg·ha^−1^) (Fig. 5). The under-representation of *L. dispar dispar* frass from field samples in 2000 was associated with our missing specific insect feeding phenologies during field sampling. Our method accounted for missing frass biomass not fully sampled in the field during the entire feeding phenology. In the case of one field plot for *C. pinus*, the discrepancy between observed and estimated frass during K-week cross-validation matched poorly for a given week. However, when we compared cumulative frass for both field-collected frass and estimated frass for this particular plot, the difference was very small (22.60 kg·ha^−1^) indicating that our method can adjust such mis-matches between feeding phenology and field frass data collection (Fig. 5).

Accurate estimation of foliage biomass loss to defoliation, when scaled to total amount of host foliage biomass, provides an estimate of annual defoliation [52], which can then be used to assess growth reduction and mortality [7,19,29]. For instance, Kulman et al. [29] in an analysis of tree growth rings revealed over 90% reduction in spring and summer growth during and two-years following heavy *C. pinus* outbreaks in Minnesota. In an another study, 100% annual defoliation of by *C. fumiferana* in two consecutive years in Maine reduced diameter growth of *A. balsamea* and *P. glauca* host by 54% and 64%, respectively (Chen et al. 2107). Given annual defoliation information, growth reduction and mortality associated with defoliators can be derived from available empirical functions [7,19], which can then be used to assess ecosystem impacts, such as carbon fluxes [14,23,30].

## Conclusion

In this study, we demonstrated an approach to integrate field-collected frass and greenfall deposition data and BioSIM simulated daily relative population data based on the feeding phenology. This approach provides the usefulness of available data (BioSIM) to fill the critical gaps missed by annual sampling efforts of defoliation which may exist in many studies but have not fully utilized yet. Improvement of field estimates of defoliation may enable better accounting of ecosystem impacts, such as tracking carbon and nutrient fluxes, by reducing uncertainly in ecosystem-based models and increasing predictive power across a wider range of scales and disciplines [23,26,51]. This approach should be suitable to calculate location- and defoliator-specific instar weighting parameters for biomass estimation in other systems. The approach is especially useful for imputing the biomass consequences of defoliation in cases where a defoliation event cannot be fully sampled through the season. Because defoliation events are episodic and often cannot be well predicted in advance, it is likely that an approach such as this will be useful for estimating seasonal totals in many field studies. As well, this approach facilitates estimation when resources may be limited for comprehensive sampling. However, we suggest that the direct use of our system-specific weighting parameters should be viewed with caution and field samplings are still recommended for model calibration. When used in conjunction with multi-spectral satellite imagery, this approach may provide a basis for translating spectral characteristics of both annual and cumulative insect disturbance to that of ecosystem impact at the landscape level [49].

## Author's contribution

**B.T., P.W., B.S.,** and **P.T.** conceived designed and interpreted results. **B.T.** led the data analysis, wrote the code and first draft. **B.S.** and **P.T.** led the field campaigns in Wisconsin and Maryland, respectively, while **J.F.** participated in data collection and assisted with BioSIM interpretation and model results. All authors have revised and edited the manuscript.

## Declaration of Competing Interest

The authors declare that they have no known competing financial interests or personal relationships that could have appeared to influence the work reported in this paper.

## Data Availability

Data will be made available on request.

## Appendix Supplementary materials

Image, application 1

## References

[1] Batzer H.O., Millers I. (1970).

[2] Barbosa P., Greenblatt J. (1979). Suitability, digestibility and assimilation of various host plants of the gypsy moth Lymantria dispar L. Oecologia.

[3] Bean J.L. (1959). Frass Size as an Indicator of Spruce Budworm Larval Instars. Ann. Entomol. Soc. Am..

[4] Bean J.L., Batzer H.O. (1957). Mean Head Width for Spruce Budworm Larval Instars in Minnesota and Associated Data1. J. Econ. Entomol..

[5] Bitterlich W. (1948). Die Winkelzahlprobe (The angle-count sample plot), Allgm. Forstu. Holzwirts. Ztg. 59: 4-5. In Forestry Abstracts (Vol. 10, p. 2314) in Flewelling, J. W. (1981). Compatible estimates of basal area and basal area growth from remeasured point samples. Forest Science.

[6] Carisey N., Bauce E. (1997). Impact of balsam fir flowering on pollen and foliage biochemistry in relation to spruce budworm growth, development and food utilization. Entomol. Exp. Appl..

[7] Chen C., Weiskittel A., Bataineh M., MacLean D.A. (2017). Evaluating the influence of varying levels of spruce budworm defoliation on annualized individual tree growth and mortality in Maine, USA and New Brunswick, Canada. For Ecol Manage.

[8] Clark D.A., Brown S., Kicklighter D.W., Chambers J.Q., Thomlinson J.R., Ni J. (2001). Measuring net primary production in forests: concepts and field methods. Ecol. Appl..

[9] Coulson R.N., Witter J.A. (1984).

[10] Crawford H.S., Jennings D.T. (1989). Predation by birds on spruce budworm Choristoneura fumiferana: functional numerical, and total responses. Ecology.

[11] Dale, Joyce, McNulty, Neilson, Ayres, Flannigan, Wotton (2001). https://academic.oup.com/bioscience/article/51/9/723/288247.

[12] Dwyer G., Dushoff J., Yee S.K. (2004). The combined effects of pathogens and predators on insect outbreaks. Nature.

[13] Dyar H.G. (1890). The Number of Molts of Lepidopterous Larvae. Psyche (New York).

[14] Dymond C.C., Neilson E.T., Stinson G., Porter K., MacLean D.A., Gray D.R., Kurz W.A. (2010). Future spruce budworm outbreak may create a carbon source in Eastern Canadian forests. Ecosystems.

[15] Esperk T., Tammaru T., Nylin S. (2007). Intraspecific variability in number of larval instars in insects. J. Econ. Entomol..

[16] Fettes J. (1950).

[17] Fletcher R.J., Hefley T.J., Robertson E.P., Zuckerberg B., McCleery R.A., Dorazio R.M (2019). A practical guide for combining data to model species distributions. Ecology.

[18] Foster J.R., Townsend P.A., Yaussy Daniel A., Hix David M., Long Robert P., Goebel P.Charles (2004). Proceedings,14th Central Hardwood Forest Conference; 2004 March 16 19; Wooster, OH. Gen. Tech. Rep. NE-316.

[19] Foster Jane R. (2017). Xylem traits, leaf longevity and growth phenology predict growth and mortality response to defoliation in northern temperate forests. Tree Physiol..

[20] Foster Jane R., Townsend P.A., Mladenoff D.J. (2013). Spatial dynamics of a gypsy moth defoliation outbreak and dependence on habitat characteristics. Landsc Ecol.

[21] Grier C.C., Lee K.M., Nadkarni N.M., Klock G.O., Edgerton P.J. (1989). Productivity of forests of the United States and its relation to soil and site factors and management practices: a review. General Technical Report PNW-GTR-222.

[22] Hansen J.D., Owens J.C., Huddleston E.W. (1981). Relation of Head Capsule Width to Instar Development in Larvae of the Range Caterpillar, Hemileuca oliviae Cockerell (Lepidoptera: saturniidae). J. Kans. Entomol. Soc..

[23] Hicke J.A., Allen C.D., Desai A.R., Dietze M.C., Hall R.J., Hogg E.H.T., Vogelmann J. (2012). Effects of biotic disturbances on forest carbon cycling in the United States and Canada. Glob Chang Biol.

[24] Higashiura Y. (1987). Larval densities and a life-table for the gypsy moth, Lymantria dispar, estimated using the head-capsule collection method. Ecol. Entomol..

[25] Jeffords M.R., Maddox J.V., McManus M.L., Webb R.E., Wieber A. (1989). Evaluation of the overwintering success of two European microsporidia inoculatively released into gypsy moth populations in Maryland. J. Invertebr. Pathol..

[26] Kennedy R.E., Ohmann J., Gregory M., Roberts H., Yang Z., Bell D.M., Seidl R. (2018). An empirical, integrated forest biomass monitoring system. Environ. Res. Lett..

[27] Kloeppel B.D., Harmon M.E., Fahey T.J. (2007). Principles and Standards for Measuring Primary Production.

[28] Kulman H.M., Hodson A.C. (1962). A Sampling Unit for the Jack-Pine Budworm, Choristoneura pinus1. J. Econ. Entomol..

[29] Kulman H.M., Hodson A.C., Duncan D.P. (1963). Distribution and effects of jack-pine budworm defoliation. Forest Science.

[30] Kurz W.A., Stinson G., Rampley G.J., Dymond C.C., Neilson E.T. (2008). Risk of natural disturbances makes future contribution of Canada's forests to the global carbon cycle highly uncertain. Proc. Natl. Acad. Sci. U.S.A..

[31] Lejeune R.R. (1950). The Effect of Jack-pine Staminate Flowers on the Size of Larvae of the Jack-pine Budworm, Choristoneura sp. Can. Entomol..

[32] Liebhold A.M., Gottschalk K.W., Muzika R.M., Montgomery M.E., Young R. (1995).

[33] Liebhold A.M., Elkinton J.S. (1988). Techniques for estimating the density of late-instar gypsy moth, Lymantria dispar (Lepidoptera: lymantriidae), populations using frass drop and frass production measurements. Environ Entomol.

[34] Leonard D.E. (1966). Differences in Development of Strains of the Gypsy Moth Porthetria dispar (L.). Bulletin of the Connecticut Agricultural Experiment Station No. 680.

[35] MacLean D.A. (2016). Impacts of insect outbreaks on tree mortality, productivity, and stand development. Can Entomol.

[36] Mattson W.J., Addy N.D. (1975). Phytophagous insects as regulators of forest primary production. Science.

[37] McCullough D.G. (2000). A review of factors affecting the population dynamics of jack pine budworm (Choristoneura pinus pinus Freeman). Popul Ecol.

[38] Mentaschi L., Besio G., Cassola F., Mazzino A. (2013). Developing and validating a forecast/hindcast system for the Mediterranean Sea. J. Coastal Res..

[39] Miller C.A. (1977). The feeding impact of spruce budworm on balsam fir. Can. J. For. Res..

[40] Miller D.A., Nichols J.D., McClintock B.T., Grant E.H.C., Bailey L.L., Weir L.A. (2011). Improving occupancy estimation when two types of observational error occur: non-detection and species misidentification. Ecology.

[41] Nealis V. (1987). The number of instars in jack pine budworm, Choristoneura pinus pinus Free.(Lepidoptera: tortricidae), and the effect of parasitism on head capsule width and development time. Can. Entomol..

[42] Nealis V.G. (1991). Parasitism in sustained and collapsing populations of the jack pine budworm, Choristoneura pinus pinus Free.(Lepidoptera: tortricidae), in Ontario, 1985–1987. Can. Entomol..

[43] Nealis V.G., Lomic P.V. (1994). Host-plant influence on the population ecology of the jack pine budworm, Choristoneura pinus (Lepidoptera: tortricidae). Ecol. Entomol..

[44] Pureswaran D.S., Roques A., Battisti A. (2018). Forest insects and climate change. Current Forestry Reports.

[45] Raupp M., Davidson J.A., Wood F.E. (1991).

[46] Régnière J., Saint-Amant R., Béchard A., Moutaoufik A. (2017).

[47] Régnière Jacques. (1990). Diapause termination and changes in thermal responses during postdiapause development in larvae of the spruce budworm, Choristoneura fumiferana. J. Insect Physiol..

[48] Régnière Jacques. (1996). Generalized approach to landscape-wide seasonal forecasting with temperature-driven simulation models. Environ Entomol.

[49] Thapa B., Wolter P.T., Sturtevant B.R., Townsend P.A. (2022). Linking remote sensing and insect defoliation biology–A cross-system comparison. Remote Sens. Environ..

[50] Thomas A.W. (1983). Proceedings: Forest Defoliator - Host Interactions: A Comparison between Gypsy Moth and Spruce Budworms.

[51] Townsend P.A., Eshleman K.N., Welcker C. (2004). Ecological Applications.

[52] Townsend P.A., Singh A., Foster J.R., Rehberg N.J., Kingdon C.C., Eshleman K.N., Seagle S.W. (2012). A general Landsat model to predict canopy defoliation in broadleaf deciduous forests. Remote Sens. Environ..

[53] Waldbauer G.P. (1968). The Consumption and Utilization of Food by Insects. Adv In Insect Phys.

[54] WDNR, Wisconsin Department of Natural Resources (2015).

[55] Williams C.A., Gu H., MacLean R., Masek J.G., Collatz G.J. (2016). Disturbance and the carbon balance of US forests: a quantitative review of impacts from harvests, fires, insects, and droughts. Glob Planet Change.

