# Supplementary material for: Linking frass and insect phenology to optimize annual forest defoliation estimation
Source: MethodsX. 2023 Feb 15;10:102075. doi: 10.1016/j.mex.2023.102075 (PMC9978851; doi:10.1016/j.mex.2023.102075)
Supplement: Supplementary file 1 [file mmc1.docx]

**Estimating seasonal defoliation impacts using frass deposition and insect phenology**

Thapa, B.^a^, Wolter, P.T.^a^, Sturtevant, B.R.^b^, Foster, J.R.^c^, & Townsend, P.A.^d^

^a^Department of Natural Resource Ecology & Management, Iowa State University, Ames, IA 50011, USA

^b^Institute for Applied Ecosystem Studies, Northern Research Station, USDA Forest Service, Rhinelander, WI 54501, USA

^c^Rubenstein School of Environment and Natural Resources, University of Vermont, Burlington, VT 05405

^d^Department of Forest and Wildlife Ecology, University of Wisconsin—Madison, 1630 Linden Drive, Madison, WI 53706, USA

# Appendix S


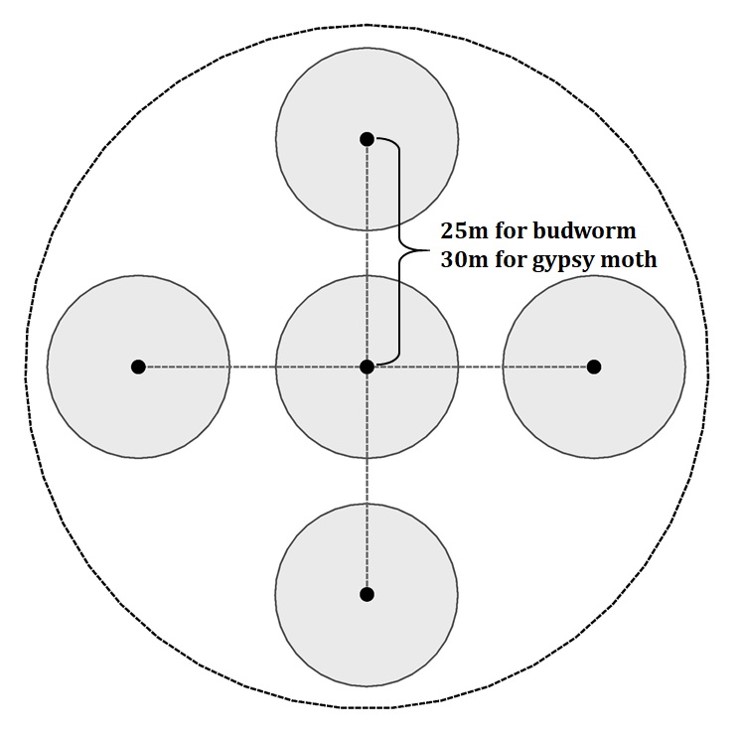


Figure S1. Five-cluster subplots layout used to collect vegetation data (basal area, diameter at breast height) and frass data. The distance between the center plot and each radial subplot is 25 m for *C. pinus* and 30 m for *L. dispar dispar* field plots.


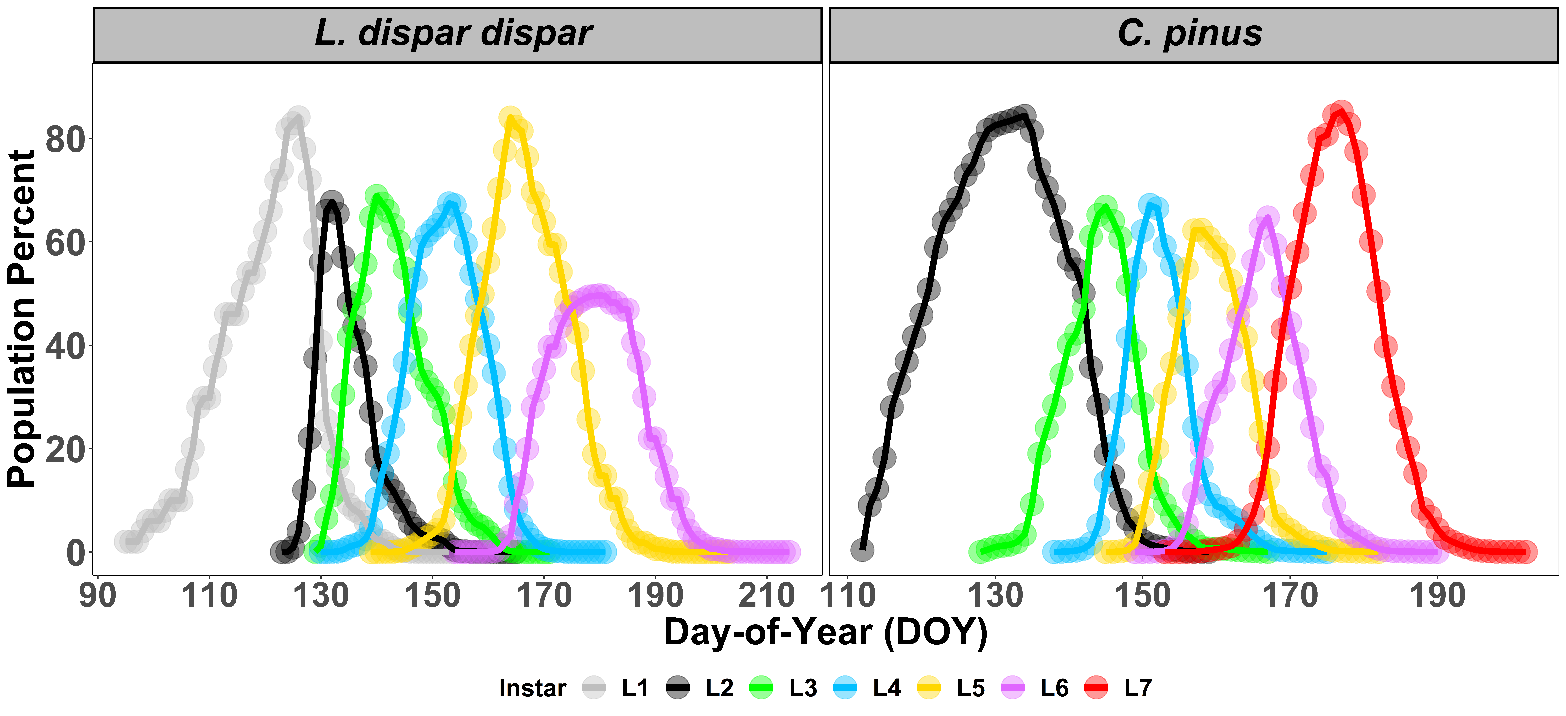


Figure S2. The distribution of population percent for *C. pinus* and *L. dispar dispar* after spring emergence (source: BioSIM V.11). Each color represents a different developmental stage, known as ‘instar’. Here, instar is denoted as ‘L’ followed by a number representing the instar stage.


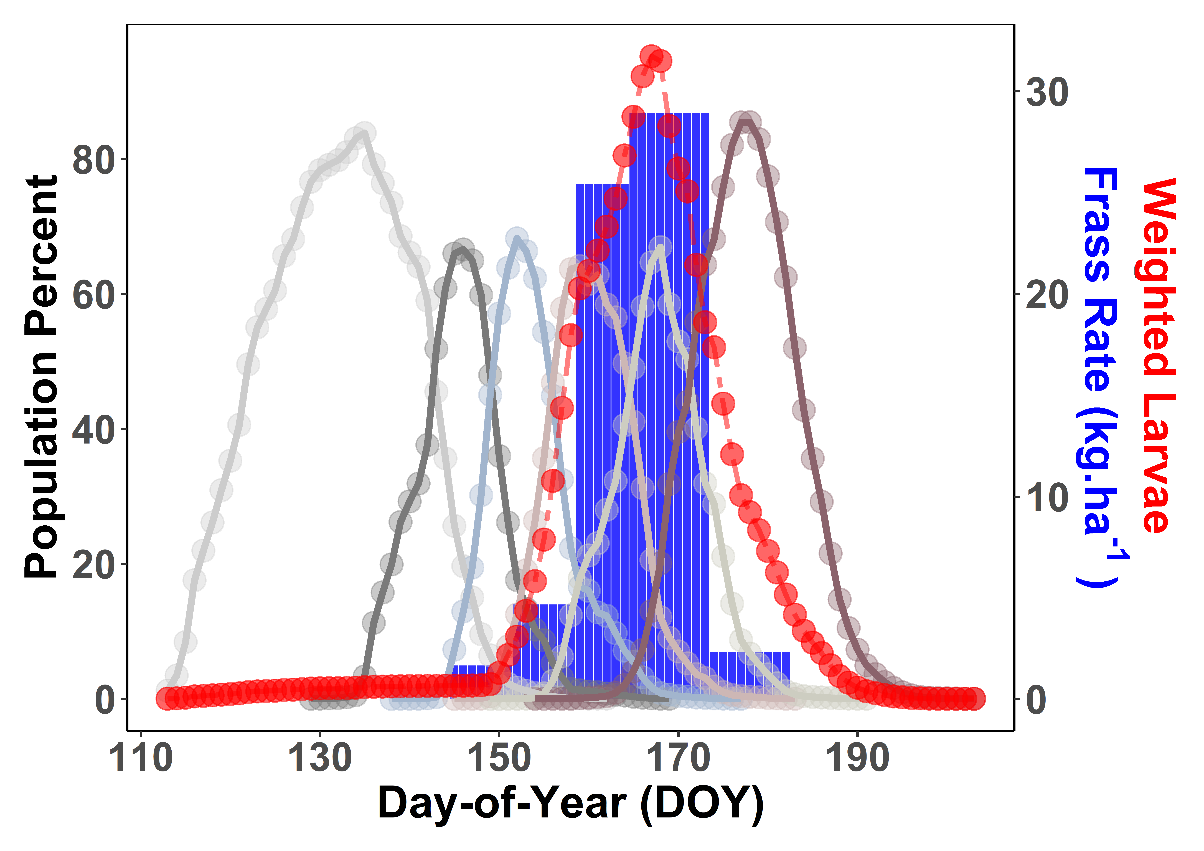


Figure S3. An example of feeding population percent data obtained from (BioSIM V. 11), where light colored points and lines represent instars 2-7, blue colored bars represent daily field-collected frass (kg⋅ha^-1^), and red colored points and line represent weighted larvae percent for each day-of-year (DOY) during the feeding season. Weighted larvae percent is the sum of population proportion for different stages of larvae (instars) scaled by instar weighting for each DOY, where instar weighting parameter (w) was estimated based on field frass collection and feeding population percentages.


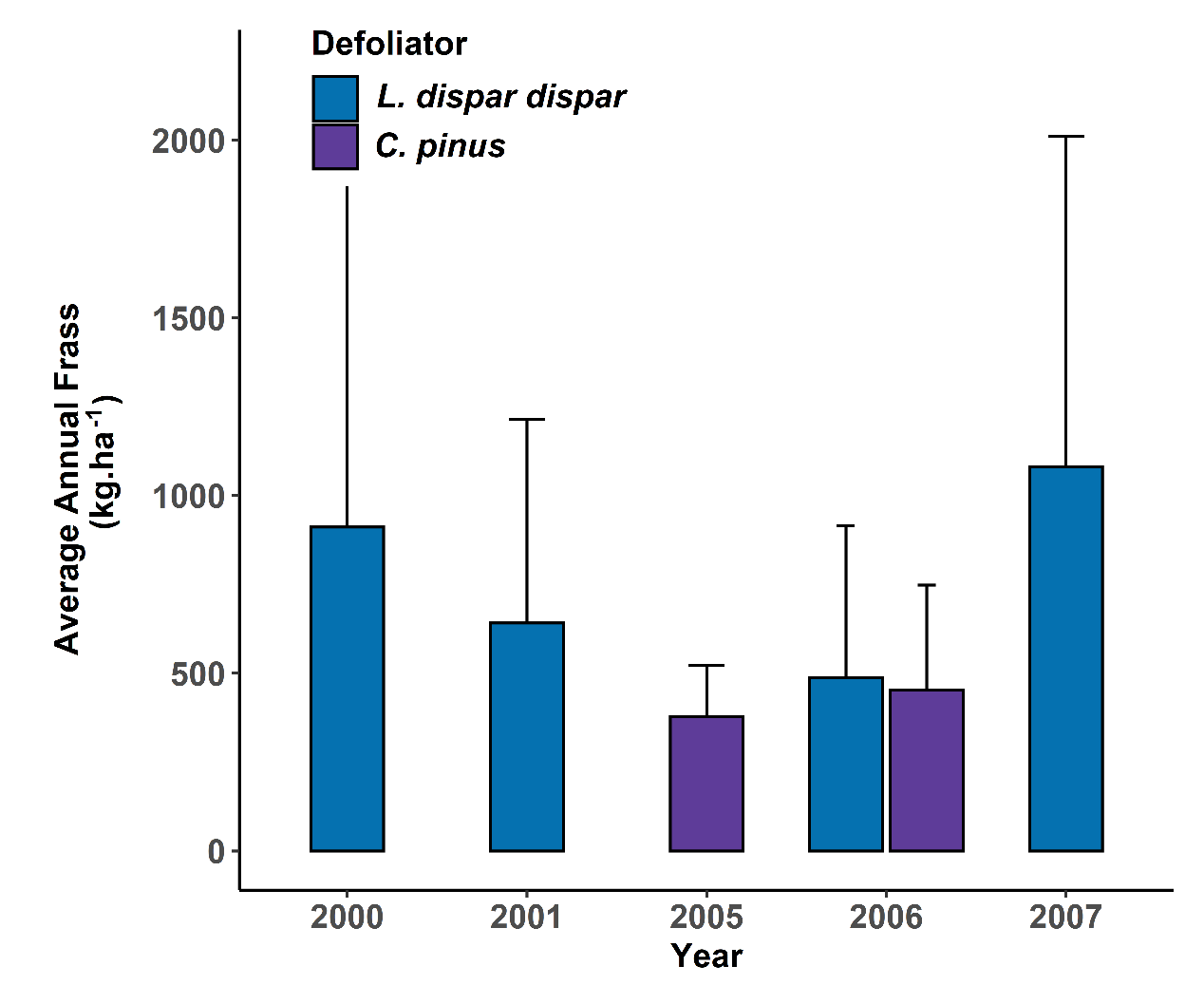


Figure S4. Distribution of average annual frass biomass (kg·ha-^1^) for each year estimated from instar weightings and then imputed for missing days in the feeding phenological window derived via BioSIM (V.11) for *C. pinus* (purple) in Wisconsin and *L. dispar dispar* (blue) in Maryland. Error bars represent one standard deviation of annual frass biomass.
